# Comparison of Abdominal Visceral Adipose Tissue Area Measured by Computed Tomography with That Estimated by Bioelectrical Impedance Analysis Method in Korean Subjects

**DOI:** 10.3390/nu7125548

**Published:** 2015-12-16

**Authors:** Dong-Hwa Lee, Kyeong Seon Park, Soyeon Ahn, Eu Jeong Ku, Kyong Yeun Jung, Yoon Ji Kim, Kyoung Min Kim, Jae Hoon Moon, Sung Hee Choi, Kyong Soo Park, Hak Chul Jang, Soo Lim

**Affiliations:** 1 Department of Internal Medicine, Seoul National University College of Medicine and Seoul National University Bundang Hospital, Seongnam 13620, Korea; roroko0902@gmail.com (D.-H.L.); yurica514@gmail.com (K.S.P.); twinstar7@naver.com (Y.J.K.); kyoungmin02@gmail.com (K.M.K.); jaemoon76@gmail.com (J.H.M.); drshchoi@snu.ac.kr (S.H.C.); janghak@snu.ac.kr (H.C.J.); 2 Division of Statistics, Medical Research Collaborating Center, Seoul National University Bundang Hospital, Seongnam 13620, Korea; ahnsoyeon@gmail.com; 3 Department of Internal Medicine, Chungbuk National University Hospital, Cheongju 28644, Korea; eujeong.ku@gmail.com; 4 Department of Internal Medicine, Eulji University Hospital, Seoul 01830, Korea; yeun6486@gmail.com; 5 Department of Internal Medicine, Seoul National University College of Medicine, Seoul 03080, Korea; ksparkmd@gmail.com

**Keywords:** visceral fat area, bioelectrical impedance, computed tomography, Korean

## Abstract

We evaluated the concordance between visceral fat area (VFA) estimated by bioelectrical impedance analysis (BIA) or computed tomography (CT) in Korean subjects with a wide range in age and body mass index (BMI). In 1006 individuals (mean age 55.2 ± 11.8 (19–87) years, mean BMI 26.0 ± 3.5 (17–46) kg/m^2^, 48.9% men), VFA quantified by CT was compared with VFA using multifrequency BIA machines within 15 days. Concordance rates were compared by age or BMI using correlation analysis, Bland-Altman plots, and intraclass correlation coefficient (ICC). Using BIA data, we established a regression formula to reflect CT-VFA. The mean VFAs by CT and BIA were 131.9 ± 57.3 cm^2^ and 110.5 ± 33.9 cm^2^, respectively (*r* = 0.605, *p* < 0.001). The mean difference was 21.4 ± 45.6 cm^2^, tending to increase with BMI. In women with BMI <25 kg/m^2^ or age <50 years, the VFAs by BIA were similar to those by CT (ICC = 0.496 in BMI <25 kg/m^2^ and ICC = 0.638 in age <50 years). However, the difference was greater in men with BMI ≥25 kg/m^2^ or age ≥50 years. Applying our formula, the difference between estimations decreased to 0.2 ± 38.2cm^2^. VFA estimated by BIA correlated well with that by CT, but a more accurate formula is needed to match CT data, particularly in older men or subjects with a high BMI.

## 1. Introduction

The prevalence of obesity has increased dramatically over the last several decades worldwide and is now a major public health problem [1]. Obesity is strongly associated with metabolic syndrome and various disorders such as type 2 diabetes mellitus, hypertension, hyperlipidemia, and cardiovascular disease. As a result, obese individuals show a higher mortality rate than normal-weight or lean counterparts [2,3]. Many studies have shown that abdominal visceral fat plays a key role in insulin resistance [4,5]. Thus, a large amount of visceral adipose tissue is a core component in subjects with metabolic syndrome [6,7].

In this context, accurate measurement of the visceral fat area (VFA) is critical in the assessment of cardiometabolic risk for each individual. Currently, abdominal computed tomography (CT) is considered one of the most accurate methods for the assessment of VFA [8]. However, CT has several drawbacks, such as high cost, the need for a CT machine, and exposure of subjects to radiation. Therefore, technical and ethical issues have been increasing regarding the use of CT for large-scale studies. Because of these limitations, various alternative methods have been tried to assess VFA [9,10,11]. Magnetic resonance imaging (MRI) provides accurate values for VFA without needing exposure to radiation but is generally more expensive than CT [11]. Furthermore, some studies indicated disagreement between CT and MRI results [12,13]. Intra-abdominal thickness measured by ultrasound (US) showed good correlation with CT [3,9]. However, US also has the limitation of inter- and intra-examiner variation.

The bioelectrical impedance analysis (BIA) method has been widely used to estimate body composition by analyzing impedance obtained when a current flows through the body [14,15]. This method is noninvasive, simple, and inexpensive. Previously, single frequency systems were used but multifrequency systems are used currently to increase accuracy [14]. So far, body composition information such as whole body fat mass, whole body fat percentage, and muscle mass measured using BIA has been used in more than 2000 studies (from a PubMed search on 15 June 2015).

There have been several experimental trials to estimate VFA in the abdomen using BIA and some of them showed good correlations between VFA estimated by BIA and that measured by CT [3,10,16]. Moreover, studies have confirmed that VFA estimated by BIA is useful for detecting metabolic impairment [15,17]. However, the number of study participants was small and their body mass index (BMI) range was not broad enough to apply their results to the general population.

Among many machines using BIA, Inbody^®^ (Inbody Co., Seoul, Korea) is one of the most frequently used machines worldwide [18,19]. The VFA estimated by BIA using Inbody^®^ showed a good correlation with VFA measured by CT in small studies [14,16]. However, there has been no large study evaluating the accuracy of the abdominal VFA obtained from the BIA method when compared with that measured by CT systemically. Here, we investigated the concordance rates between VFA estimated by BIA and that measured by CT in a large number of people with wide ranges in age and BMI values.

## 2. Materials and Methods

### 2.1. Subjects

We recruited 1006 consecutive Korean subjects, 19–87 years of age (492 men, 514 women) who visited Seoul National University Bundang Hospital (SNUBH) for their medical checkup in the period March 2007 to June 2015. Those who had a malignancy, a history of major surgery on their extremities, chronic kidney disease stage IV or renal replacement therapy, liver cirrhosis with ascites, heart failure with peripheral edema, or severe hypothyroidism were excluded. Patients with fever resulting from an active infection or inflammation, those receiving systemic steroid treatment, and those suffering severe dehydration such as from uncontrolled diabetes mellitus were also excluded. The study protocol was approved by the ethics committee of SNUBH (SNUBH IRB#B-1503/292-106) and all participants provided written informed consent.

### 2.2. Anthropometrics

Height and body weight were measured by standard protocols at the time of Inbody^®^ testing. The BMI was calculated by dividing body weight (in kg) by the square of the height (in m). Waist circumference was measured in the standing position at the midpoint between the lateral iliac crest and the lowest rib at the end of expiration while the subject was breathing gently. Blood pressure was measured using an automatic blood pressure measurement device. Each subject rested for at least 5 min prior to blood pressure measurements while sitting in a chair with both feet flat on the floor and both arms supported at the level of the heart.

### 2.3. Biochemical Tests

Fasting blood samples were obtained in the morning after a 12-h fast including any medication. The glycated hemoglobin (A1c) level was measured by affinity chromatography (Bio-Rad Laboratories, Hercules, CA, USA). A complete blood cell count analysis was performed using Sysmex XE-2100 (Sysmex, Mundelein, IL, USA). Fasting plasma concentrations of total cholesterol, triglycerides, high-density lipoprotein (HDL)-cholesterol, low-density lipoprotein (LDL)-cholesterol, and serum creatinine were measured on a Hitachi 747 chemistry analyzer (Hitachi, Tokyo, Japan). Estimated glomerular filtration rate (eGFR) was calculated using the Modification of Diet in Renal Disease equation [20]. Serum aspartate aminotransferase (AST) and alanine aminotransferase (ALT) were measured with an autoanalyzer (TBA-200FR, Toshiba, Tokyo, Japan).

### 2.4. Abdominal VFA Estimation by BIA

Abdominal VFA was estimated using three multifrequency BIA machines (Inbody720^®^; Inbody Co.) for each individual, in a fasting state on the same day as their blood test. The study participants were requested to refrain from smoking, drinking alcohol, and strenuous exercise for 48 h prior to measurement. After the subjects had been guided to stand on the platform of the device, age and gender information were entered into the machine. After confirming that the subject was standing correctly with both arms apart from the body and both feet on the right spots on the platform, a supervisor pushed the start button to perform assessment. Both hands were held at a 45° angle away from the body. The device scans at X-scan uses 1, 5, 50, 250, 500 kHz, and 1 MHz frequencies to analyze intracellular and extracellular fluid values and water content.

### 2.5. Abdominal VFA Measurement Using CT

Images of VFA at the umbilical level were taken from CT scans with each individual in a fasting state. A cross-sectional abdominal contour was estimated by outlining the skin manually with a graph pen through the muscular structures and lumbar spines. The area between −250 Hounsfield units (HU) and −50 HU pixels was calculated automatically using dedicated software (Rapidia, 3DMED, Seoul, Korea). The CT scan was performed within 15 days of the BIA measurement.

### 2.6. Statistical Analysis

Data are presented as the mean ± standard deviation (SD), and *p* < 0.05 was considered significant. The Kolmogorov-Smirnov Goodness of Fit test was used to confirm the normal distribution of the key variables. Paired *t* test and intraclass correlation coefficient (ICC) for absolute agreement were used to compare the difference between two methods. Pearson’s correlation coefficient was used to investigate any correlations between VFA measured by CT and estimated by BIA. Analysis of the comparability and agreement levels between the two methods was conducted using the Bland-Altman method. Fisher’s r to z transformation was used to compare the correlation coefficients among subgroups according to the clinical characteristics.

To estimate VFA from BIA more accurately for referencing against VFA values by CT, multiple linear regression models were developed for each gender using age, BMI, and VFA value obtained from the BIA machine. These independent factors were chosen based on clinical judgments. For continuous factors such as VFA estimated by BIA and BMI, a linear relationship was set after investigating nonlinearity through restricted cubic splines. Potential interaction effects were examined using the backward stepwise selection method and the final model was derived based on the Akaike Information Criterion (AIC) and Bayesian Information Criterion [21]. Calibration was performed using a graphical plot of either the apparent or the bias-corrected predictions on the *X*-axis and the observations on the *Y*-axis. All statistical analyses were performed using SPSS for Windows version 22.0 (IBM Corp., Armonk, NY, USA) and R program version 3.2.0 (R Foundation for Statistical Computing, Vienna, Austria).

## 3. Results

### 3.1. Baseline Clinical Characteristics of the Study Populations

Table 1 summarizes the baseline characteristics of the study population (*n* = 1006). The mean ± SD (range) age and BMI of the population were 55.2 ± 11.8 (19–87) years and 26.0 ± 3.5 (17–46) kg/m^2^, respectively. Men had a greater mean waist circumference than women by about 5 cm. Men comprised significantly higher percentages of current or ex-smokers (*p* < 0.001) and alcohol drinkers (*p* < 0.001).

**Table 1:** Anthropometric and biochemical characteristics and comorbidity of the study populations.

	All	Men	Women	*p
	(*n* = 1006)	(*n* = 492)	(*n* = 514)	
Age (years)	55.2 ± 11.8	53.7 ± 11.9	56.7 ± 11.5	<0.001
Height (cm)	163.4 ± 8.7	169.6 ± 6.1	157.4 ± 6.2	<0.001
Weight (kg)	69.0 ± 12.4	74.8 ± 11.9	63.5 ± 10.1	<0.001
BMI (kg/m^2^)	26.0 ± 3.5	26.1 ± 3.4	25.8 ± 3.6	0.193
WC (cm)	89.2 ± 9.5	91.8 ± 8.7	86.7 ± 9.5	<0.001
SBP (mmHg)	129.6 ± 15.2	130.5 ± 16.0	128.7 ± 14.4	0.059
DBP (mmHg)	78.1 ± 10.8	79.5 ± 10.9	76.7 ± 10.6	<0.001
***Laboratory findings***				
FPG (mg/dL)	138.1 ± 48.9	143.6 ± 49.4	132.2 ± 47.8	0.001
A1c (%)	7.1 ± 1.8	7.3 ± 1.8	7.0 ± 1.8	0.018
WBC (10^3^/μL)	6.65 ± 2.13	7.02 ± 2.28	6.32 ± 1.93	<0.001
Hemoglobin (g/dL)	14.2 ± 1.7	15.1 ± 1.5	13.4 ± 1.4	<0.001
Hematocrit (%)	42.7 ± 4.5	45.1 ± 4.0	40.6 ± 3.7	<0.001
Platelet (10^3^/μL)	241.1 ± 58.2	227.6 ± 57.0	253.1 ± 56.7	<0.001
Total cholesterol (mg/dL)	200.9 ± 41.6	197.5 ± 40.8	204.2 ± 42.0	0.011
Triglycerides (mg/dL)	156.2 ± 94.7	165.5 ± 97.3	147.7 ± 91.5	0.005
HDL-cholesterol (mg/dL)	52.5 ± 14.4	49.9 ± 15.5	55.0 ± 12.9	<0.001
LDL-cholesterol (mg/dL)	109.2 ± 31.1	110.4 ± 31.0	108.2 ± 31.3	0.281
BUN (mg/dL)	14.7 ± 4.6	15.1 ± 4.4	14.3 ± 4.7	0.003
Cr (mg/dL)	0.97 ± 0.21	1.07 ± 0.19	0.88 ± 0.18	<0.001
eGFR (mL/min/1.73 m^2^)	76.0 ± 16.0	78.9 ± 16.3	73.2 ± 15.2	<0.001
AST (IU/L)	27.3 ± 17.3	29.7 ± 21.2	25.0 ± 12.0	<0.001
ALT (IU/L)	32.8 ± 28.7	38.3 ± 35.2	27.4 ± 19.1	<0.001
***Fat area at umbilicus level measured by CT***				
VFA by CT (cm^2^)	131.9 ± 57.3	145.1 ± 60.4	119.3 ± 51.1	<0.001
SFA by CT (cm^2^)	182.2 ± 83.9	148.2 ± 73.4	214.7 ± 80.5	<0.001
***Body composition by BIA***				
Total body water (L)	35.6 ± 7.1	41.0 ± 5.1	30.4 ± 4.3	<0.001
Lean body mass (kg)	45.7 ± 9.2	52.7 ± 6.6	39.0 ± 5.8	<0.001
Whole body fat mass (kg)	21.2 ± 7.5	19.8 ± 7.7	22.5 ± 7.0	<0.001
Whole body fat percent (%)	30.3 ± 8.1	25.6 ± 6.4	34.8 ± 6.8	<0.001
VFA by BIA (cm^2^)	110.5 ± 33.9	106.9 ± 34.9	113.9 ± 32.6	0.001
***Lifestyles, %***				
Smoking				
non/ex-/current	61.8/18.7/19.5	27.3/35.3/37.4	95.1/2.8/2.1	<0.001
Alcohol				
non/light to moderate/heavy	60.3/31.2/8.5	35.8/49.9/14.3	84.3/12.9/2.8	<0.001
Exercise				
Regular/irregular or Non	57.3/42.7	59.7/40.3	54.9/45.1	0.002
***Comorbidity, n (%)***				
Diabetes mellitus	664 (66.0)	358 (72.8)	306 (59.5)	<0.001
Hypertension	408 (40.8)	199 (40.7)	209 (40.8)	0.968
Dyslipidemia	441 (45.1)	212 (44.4)	229 (45.8)	0.649
***Medications, n (%)***				
Diuretics	122 (12.1)	59 (12.0)	63 (12.3)	0.898
Thiazolidinedione	47 (4.7)	26 (5.3)	21 (4.1)	0.368

Data are expressed as the mean ± SD. BMI, body mass index; WC, waist circumference; SBP, systolic blood pressure; DBP, diastolic blood pressure; WBC, white blood cell; FPG, fasting plasma glucose; LDL, low-density lipoprotein; HDL, high-density lipoprotein; BUN, blood urea nitrogen; Cr, creatinine; VFA, visceral fat area; SFA, subcutaneous fat area. *p values by Student’s *t* test between men and women.

In this population, VFAs (mean ± SD (ranges)) at the umbilicus level measured by CT and by BIA were 131.9 ± 57.3 (11.0–358.9) cm^2^ and 110.5 ± 33.9 (24.2–246.6) cm^2^, respectively. The mean VFA measured by CT (CT-VFA) was significantly larger in men than in women (145.1 ± 60.4 *vs.* 119.3 ± 51.1 cm^2^, *p* < 0.001). In contrast, the mean VFA by BIA (BIA-VFA) in women was significantly larger than in men (113.9 ± 32.6 *vs.* 106.9 ± 34.9 cm^2^, *p* = 0.001).

### 3.2. Associations between VFAs Measured by CT and BIA

**Figure 1:**
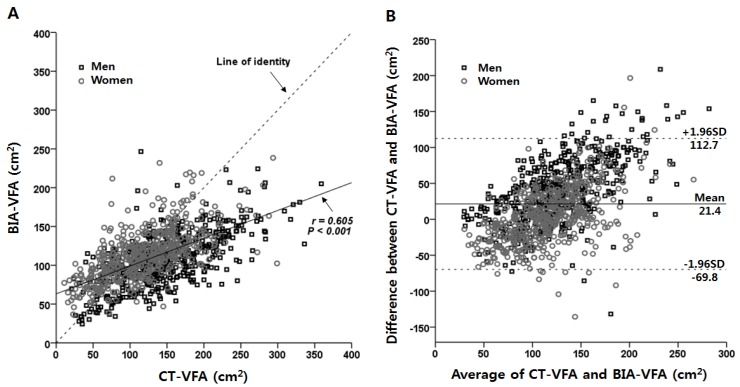
Correlation between VFA measured by CT and VFA estimated by BIA (**A**) and Bland-Altman plot for comparing the two methods (**B**).

Using Pearson’s correlation test for comparing the two techniques, the BIA-VFA showed a significant positive correlation with CT-VFA (*r* = 0.605, *p* < 0.001) (Figure 1A). The Bland-Altman method of comparison between CT-VFAs and BIA-VFAs showed a mean bias of 21.4 ± 45.6 cm^2^ (Figure 1B and Table 2), indicating that the BIA-VFA was smaller than the CT-VFA by 21.4 cm^2^. A Bland-Altman plot showed that the BIA method underestimated abdominal VFA in those subjects with a high CT-VFA (≥100 cm^2^). In contrast, it tended to overestimate VFA in those having a lower CT-VFA (<100 cm^2^).

**Table 2:** Mean differences of VFAs observed by CT and BIA according to the gender, body mass index, and age groups.

	N	CT-VFA (cm^2^)	BIA-VFA (cm^2^)	CT-VFA-BIA-VFA (cm^2^)	*p	†*p*	ICC
Total	1006	131.9 ± 57.3	110.5 ± 33.9	21.4 ± 45.6	<0.001		0.481
Gender						<0.001	
Men	492	145.1 ± 60.4	106.9 ± 34.9	38.2 ± 45.9	<0.001		0.438
Women	512	119.3 ± 51.1	113.9 ± 32.6	5.4 ± 39.2	0.002		0.577
BMI (kg/m^2^)						<0.001	
<20	23	53.5 ± 24.1	67.6 ± 16.4	−14.1 ± 29.2	0.031		−0.008
20–22.9	161	81.1 ± 37.2	77.8 ± 22.9	3.2 ± 33.3	0.218		0.417
23–24.9	232	113.3 ± 42.7	98.3 ± 23.5	15.0 ± 39.3	<0.001		0.319
25–26.9	248	138.6 ± 44.3	108.9 ± 19.4	29.7 ± 43.5	<0.001		0.140
27–29.9	223	158.0 ± 52.0	125.3 ± 22.8	32.8 ± 48.8	<0.001		0.196
≥30	119	189.3 ± 55.9	162.1 ± 31.4	27.2 ± 58.4	<0.001		0.145
Age (years)						0.050	
19–39	95	107.8 ± 64.8	99.3 ± 42.8	8.5 ± 52.2	0.115		0.544
40–49	205	123.5 ± 50.4	101.8 ± 33.8	21.7 ± 40.4	<0.001		0.496
50–59	314	131.3 ± 55.1	106.8 ± 30.3	24.4 ± 44.7	<0.001		0.430
60–69	291	138.1 ± 55.4	116.9 ± 30.5	21.2 ± 45.3	<0.001		0.438
≥70	101	155.8 ± 64.0	131.2 ± 32.9	24.6 ± 51.4	<0.001		0.441

Data are expressed as the mean ± SD. *p values by paired *t* test between CT-VFA and BIA-VFA. †*p* values by One-way ANOVA for changes across groups.

In the subgroup analyses according to clinical features, such as having anemia with a 12 g/dL cutoff of hemoglobin, kidney function using an eGFR of 60 mL/min/1.73 m^2^, liver function using an AST or ALT of 40 IU/L, diabetes mellitus or glycemic control (A1c = 8.0%), and diuretics and thiazolidinedione medications, there were no significant differences in correlation coefficients between groups from Fisher z-tests (Table 3).

**Table 3:** Pearson correlation between VFA measured by CT and VFA estimated by BIA in subgroups according to clinical features.

		*n*	*r*	*p	†*p*
**Anemia**	Hb ≥ 12 g/dL	662	0.652	<0.001	0.219
	Hb < 12 g/dL	37	0.510	0.001	
**Kidney Function**	eGFR ≥ 60 mL/min/1.73 m^2^	740	0.630	<0.001	0.327
	eGFR < 60 mL/min/1.73 m^2^	103	0.563	<0.001	
**Liver Function**	ALT ≥ 40 IU/L	227	0.607	<0.001	0.569
	ALT < 40 IU/L	771	0.579	<0.001	
	AST ≥ 40 IU/L	111	0.503	<0.001	0.144
	AST < 40 IU/L	887	0.606	<0.001	
**Diabetes Mellitus**	DM (−)	342	0.625	<0.001	0.516
	DM (+)	664	0.598	<0.001	
	HbA1c < 8%	428	0.574	<0.001	0.223
	HbA1c ≥ 8%	236	0.637	<0.001	
**Medications**	Diuretics (−) and Thiazolidinedione (−)	823	0.608	<0.001	
	Thiazolidinedione (+)	47	0.659	<0.001	0.484
	Diuretics (+)	122	0.564	<0.001	0.430

*p values by persons correlation analysis between CT-VFA and BIA-VFA. *^†^p* values by Fisher z-test between correlation coefficients.

### 3.3. Agreement Levels between VFAs by CT and BIA According to Gender, BMI, and Age

To determine whether the concordance rates between CT-VFAs and BIA-VFAs differed by gender, BMI, or age, we divided the study populations into subgroups according to gender, BMI (<20, 20–22.9, 23–24.9, 25–26.9, 27–29.9 and ≥30 kg/m^2^), and age (19–39, 40–49, 50–59, 60–69 and ≥70 years).

**Figure 2:**
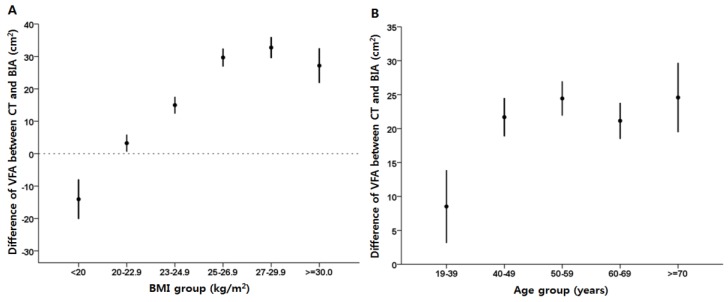
Differences in VFA between CT and BIA methods according to BMI (**A**) and subjects age (**B**).

The BIA-VFA was lower than the CT-VFA in both genders (Table 2). The mean difference was larger in men than in women (38.2 ± 45.9 *vs.* 5.4 ± 39.2 cm^2^, *p* < 0.001). Among the BMI categories, BIA-VFAs were larger than CT-VFAs in the BMI <20 kg/m^2^ group. In the groups with BMI ≥20 kg/m^2^, BIA-VFAs were smaller than CT-VFAs with an increasing trend in the mean difference as BMI increased up to 30 kg/m^2^. The mean differences in VFA estimates tended to increase along with BMI except in subjects with BMI ≥30 kg/m^2^ (Figure 2A and Table 2). BIA-VFAs were smaller than CT-VFAs in all age groups. The mean difference between BIA-VFA and CT-VFA tended to increase with age except for the group of aged 60–69 years (Figure 2B and Table 2).

The ICC value between the two methods was 0.481 in the total population indicating overall good reliability. Similar to the mean difference in VFA values, the ICC value was higher in women than in men. The ICC value decreased with increasing BMI, while this trend was not found in the increasing age categories (Table 2).

### 3.4. Subgroup Comparison of CT-VFAs and BIA-VFAs According to Age and BMI Categories by Gender

Because body composition is fundamentally different between men and women, we analyzed the data further according to gender with the study population divided into two subcategories: nonobese (BMI < 25 kg/m^2^) *vs.* overweight or obese (BMI ≥ 25 kg/m^2^); and younger (age < 50 years) *vs.* older (age ≥ 50 years). In men, BIA-VFAs were significantly smaller than CT-VFAs in both age groups (both *p* < 0.001). In women with BMI ≥ 25 kg/m^2^ or age ≥ 50 years, BIA-VFAs were also significantly smaller than CT-VFAs (*p* < 0.001 for both groups). In women with BMI <25 kg/m^2^ or age <50 years, BIA-VFAs were similar to CT-VFAs (mean differences were −1.4 ± 33.8 cm^2^ in the BMI <25 kg/m^2^ group and −4.9 ± 36.4 cm^2^ in the age <50 years group; both *p* > 0.05). The ICC values showed a similar tendency to the mean differences in VFAs. The highest ICC value was 0.638 in women with age <50 years (Table 4).

**Table 4:** Subgroup analyses of mean differences between CT-VFA and BIA-VFA by gender.

	*n*	CT-VFA (cm^2^)	BIA-VFA (cm^2^)	CT-VFA–BIA-VFA (cm^2^)	*p*	ICC
**Men**						
BMI (kg/m^2^)						
<25	188	105.4 ± 45.0	84.2 ± 28.0	21.2 ± 37.8	<0.001	0.424
≥ 25	304	169.6 ± 55.6	120.9 ± 31.2	48.8 ± 47.3	<0.001	0.285
Age (years)						
<50	175	132.4 ± 56.7	98.9 ± 36.9	33.5 ± 43.4	<0.001	0.472
≥50	317	152.1 ± 61.4	111.3 ± 33.0	40.8 ± 47.0	<0.001	0.407
**Women**						
BMI (kg/m^2^)						
<25	228	91.0 ± 42.0	92.4 ± 22.4	−1.4 ± 33.8	0.539	0.496
≥25	286	141.8 ± 46.3	131.1 ± 29.1	10.7 ± 42.3	<0.001	0.387
Age (years)						
<50	125	99.1 ± 48.3	104.0 ± 36.8	−4.9 ± 36.4	0.135	0.638
≥50	389	125.8 ± 50.3	117.1 ± 30.5	8.7 ± 39.6	<0.001	0.537

Data are expressed as the mean ± SD; *p* values by paired *t* test between CT-VFA and BIA-VFA.

### 3.5. New Formula to Predict CT-VFA Using BIA-VFA Data

**Figure 3:**
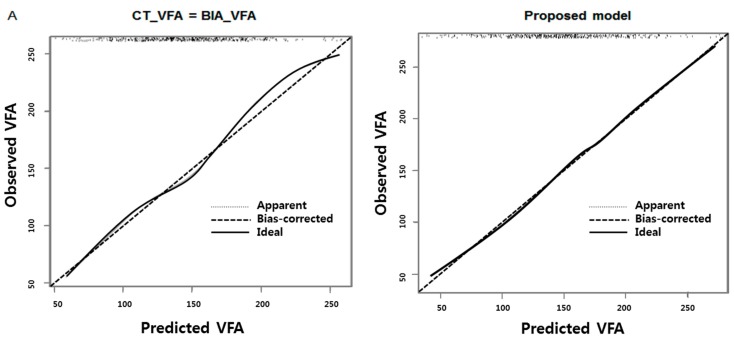

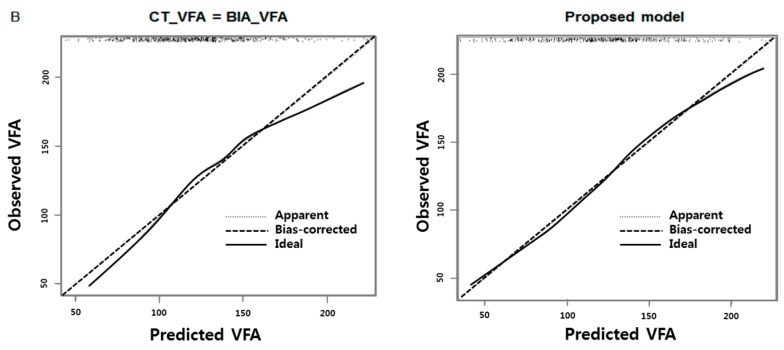
Calibration plots of prediction model in men (**A**) and women (**B**).

After stepwise backward selection with a minimal AIC, the same sets of main variables and interaction effects were retained in the final models in men and women: BIA-VFA, age, BMI, WC, BIA-VFA × BMI, and age × BMI. Compared with a univariate prediction model with BIA-VFA only, the AIC decreased from 5146.9 to 4697.0 in men and from 5193.0 to 4330.1 in women, resulting in an improvement in the agreement between observations and predictions (Figure 3).

The final models were derived as: y = (−184.51) + 1.11 × BIA-VFA + (−1.49) × Age + 2.10 × BMI + 2.03 × WC + (−0.02) × BIA-VFA × BMI + 0.08 × age × BMI for men; and y = (−142.77) + 1.40 × BIA-VFA + (−1.29) × Age + (–0.98) × BMI + 2.14 × WC + (−0.03) × BIA-VFA × BMI + 0.07 × age × BMI for women (Table 5).

**Table 5:** Multivariate regression models for prediction of VFA measured by CT according to gender.

	Men			Women		
	Coefficient	95% CI		Coefficient	95% CI	
		Lower	Upper		Lower	Upper
Intercept	−184.51	−323.06	−45.95	−142.77	−277.24	−8.30
BIA-VFA	1.11	0.46	1.76	1.40	0.72	2.08
Age	−1.49	−3.61	0.63	−1.29	−3.68	1.10
BMI	2.10	−3.28	7.47	−0.98	−6.49	4.54
WC	2.03	1.33	2.72	2.14	1.55	2.73
BIA-VFA*BMI	−0.02	−0.04	0.01	−0.03	−0.05	−0.01
Age*BMI	0.08	−0.01	0.16	0.07	−0.02	0.17

## 4. Discussion

In this study, abdominal VFAs estimated by BIA were significantly correlated with those measured by CT (*r* = 0.605, *p* < 0.001). However, the concordance rate between BIA and CT in abdominal VFAs differed according to BMI and age groups by gender. The concordance rate was quite high in women (mean difference = 5.4 ± 39.2 cm^2^ and ICC = 0.577) whereas there was a larger difference in men (mean difference = 38.2 ± 45.9 cm^2^ and ICC = 0.438). Differences in the VFAs between the two methods changed with BMI. The BIA method used in this study overestimated VFAs in those with BMI < 20 kg/m^2^ and underestimated VFAs in those with BMI ≥ 20 kg/m^2^ with a dose-dependent relationship. Among the age groups, the BIA method estimated abdominal VFAs well in the age range of 19–39 years whereas it underestimated the CT-VFAs in those aged over 40 years.

Various methods are used to assess fat accumulation in the abdomen, particularly in the visceral area. BMI is the most common method for estimating body fat, and previous studies demonstrated its correlation with abdominal visceral fat levels [22]. Waist circumference and waist–hip ratio are also commonly used measures for the prediction of intra-abdominal fat deposition because these methods are easy to apply [23,24]. However, BMI reflects not only the visceral fat, but also the total body fat amount, so there is a lack of concordance with abdominal VFA measured by CT [3,25]. Using the waist circumference also has limitations such as inter- and intra-examiner variations [26]. Indeed, the amount of visceral adipose tissue predicted by BMI or other anthropometric parameters is underestimated with increasing BMI [9,27]. Furthermore, waist circumference is correlated better with subcutaneous fat than with visceral fat [28].

The CT technique has been proposed as the gold standard method to quantify visceral fat since 1990 [29], and has been used widely to assess visceral adiposity in many clinical and experimental studies [30,31,32]. The Japan Society for the Study of Obesity originally proposed ≥100 cm^2^ as a VFA cutoff value for defining visceral obesity, which is one of the essential diagnostic criteria for metabolic syndrome in Japan [33]. However, CT exposes subjects to radiation and the equipment is expensive. Therefore, CT is not suitable for studies with large numbers of participants or periodical measurements. In contrast, BIA has several advantages: subjects are not exposed to radiation, and BIA machines are relatively cheap and portable, which enables clinics to use them more freely [34]. BIA measures the impedance of the human body electrically and can be used to estimate human body compositions. However, it is influenced by several factors and conditions [35]. Therefore, standardized conditions with regard to body position, previous exercise, dietary intake, and skin temperature must be considered. Age is also known to be an important factor. A number of BIA equations developed in young subjects result in large biases when applied to older subjects [36]. Furthermore, subjects with extreme BMI values or abnormal tissue hydration and suffering edema or taking drugs that affect water balance also influence the results of BIA [35].

BIA has extended its use to VFA estimation. Previous studies have demonstrated that VFA estimated by BIA is well correlated to that measured by CT [3,10,25,37]. One study showed that BIA was clinically useful to detect an excessive accumulation of visceral fat in medical checkups [17]. Moreover, BIA is more sensitive than other methods in detecting weight change during weight reduction therapy [25,38]. Here, multifrequency BIA underestimated abdominal VFAs compared with CT scans except for women with BMI <25 kg/m^2^ or age <50 years. This finding was consistent with that of another study showing a difference in VFA estimated by BIA compared with that measured by CT according to gender, BMI, or age [3]. To obtain more precise VFA values, we generated a new formula using regression models including VFA estimated by BIA, age, BMI, and WC for each gender. The new formula could generate more accurate VFAs than those obtained originally from the BIA machine.

Our study has several strengths. To our knowledge, this is the largest study covering a wide spectrum of age and BMI values. In addition, various comorbidities such as diabetes mellitus, hypertension, and dyslipidemia, lifestyles such as smoking status, alcohol consumption, and exercise habit, and laboratory findings were adjusted in the analysis.

**Figure 4:**
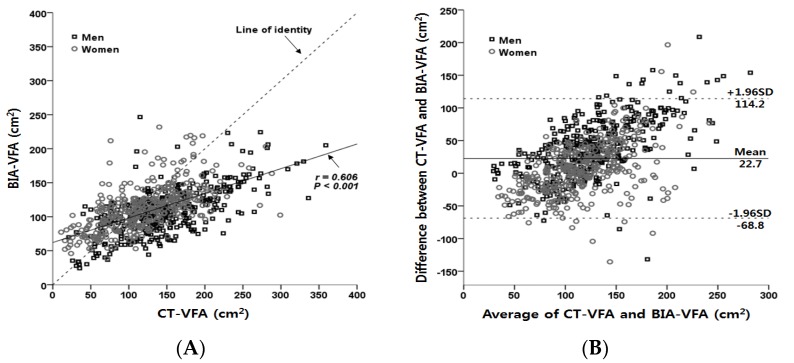
Correlation between VFA measured by CT and VFA estimated by BIA (**A**) and Bland-Altman plot for comparison between two methods (**B**) in subjects who performed CT and BIA within seven days.

There are also a number of limitations. First, not all of the BIA and CT measurements were performed on the same day. However, when we performed a subgroup analysis using those subjects who underwent BIA and CT measurements within 7 days (*n* = 722), similar results were obtained (Figure 4). Second, this study was conducted in a single Asian ethnic group, so it cannot be generalized to other ethnic groups.

## 5. Conclusions

Our study has shown that VFA estimated by multifrequency BIA was significantly correlated with VFA measured directly by CT. The BIA method tends to underestimate VFA with increase in BMI or age except for women aged < 50 years or in those with a BMI < 25 kg/m^2^. The application of a slightly modified formula using simply age, BMI, original VFA, and its interaction terms made the VFA values more accurate for the reference values obtained from CT scans. Further investigation of BIA technology is warranted for better estimating visceral fat depots in the abdomen.
